# Human amnion epithelial cells modulate the inflammatory response to ventilation in preterm lambs

**DOI:** 10.1371/journal.pone.0173572

**Published:** 2017-03-27

**Authors:** Jacqueline M. Melville, Courtney A. McDonald, Robert J. Bischof, Graeme R. Polglase, Rebecca Lim, Euan M. Wallace, Graham Jenkin, Timothy J. Moss

**Affiliations:** 1 The Ritchie Centre, Hudson Institute of Medical Research, Clayton, Victoria, Australia; 2 Department of Obstetrics and Gynaecology, Monash University, Clayton, Victoria, Australia; Shanghai Jiao Tong University, CHINA

## Abstract

Ventilation of preterm neonates causes pulmonary inflammation that can contribute to lung injury, propagate systemically and result in long-term disease. Modulation of this initial response may reduce lung injury and its sequelae. We aimed to determine the effect of human amnion epithelial cells (hAECs) on immune activation and lung injury in preterm neonatal lambs. Preterm lambs received intratracheal hAECs (90x10^6^) or vehicle, prior to 2 h of mechanical ventilation. Within 5 min of ventilation onset, lambs also received intravenous hAECs (90x10^6^) or vehicle. Lung histology, bronchoalveolar lavage (BAL) cell phenotypes, and cytokine profiles were examined after 2 h of ventilation, and in unventilated controls. Histological indices of lung injury were higher than control, in vehicle-treated ventilated lambs but not in hAEC-treated ventilated lambs. Ventilation-induced pulmonary leukocyte recruitment was greater in hAEC-treated lambs than in vehicle-treated lambs. Lung IL-1β and IL-6 mRNA expression was higher in vehicle- and hAEC-treated ventilated lambs than in controls but IL-8 mRNA levels were greater than control only in vehicle-treated ventilated lambs. Numbers of CD44^+^ and CD21^+^ lymphocytes and macrophages from the lungs were altered in vehicle- and hAEC-treated ventilated lambs. Numbers of CD8^+^ macrophages were lower in hAEC-treated ventilated lambs than in vehicle-treated ventilated lambs. Indices of systemic inflammation were not different between vehicle- and hAEC-treated lambs. Human amnion epithelial cells modulate the pulmonary inflammatory response to ventilation in preterm lambs, and reduce acute lung injury. Immunomodulatory effects of hAECs reduce lung injury in preterm neonates and may protect against longer-term respiratory disease.

## Introduction

Assisted or mechanical ventilation at birth is required by about 1 in 10 neonates [1], and is often provided in the form of intermittent positive pressure ventilation [2]. Such ventilation, while needed, causes inadvertent lung injury and long-term respiratory disease in some babies [3]. Such injury may contribute to development of bronchopulmonary dysplasia (BPD) [4].

Mechanical ventilation can result in airway inflammation and elevated pulmonary mRNA expression of inflammatory mediators, including serum amyloid A3 (SAA-3), interleukin (IL)-1β and IL-6, within 1 h of ventilation [5, 6]. Activation of circulating CD4^+^ and CD8^+^ T cells occurs in preterm infants with BPD [7], however, the absolute lymphocyte number is decreased, owing to a decrease in circulating CD4^+^ cells [8]. This initial inflammatory response to ventilation may influence development of lung injury [9, 10]. Prevention or attenuation of early detrimental responses to mechanical ventilation, through immunomodulation, may therefore reduce the risk of development of BPD [11].

One potential immunomodulatory therapy for BPD is administration of stem cell–like human amnion epithelial cells (hAECs). Epithelial cells of the amnion can differentiate into ectodermal, mesodermal and endodermal lineages [12–14], and into lung epithelial–like cells *in vitro* [15, 16] and *in vivo* [17]. Administration of hAECs reduces inflammatory gene expression in lung tissue and prevent inflammation-induced changes in fetal lung development induced by either intra-amniotic injection of lipopolysaccharide [18] or a 12-h period of mechanical ventilation *in utero* [17] in sheep. Human AECs can also moderate abnormal lung development in hyperoxic neonatal mice [19], but their ability to influence ventilation-induced lung inflammation and injury in neonates has not been examined.

We hypothesised that administration of hAECs to ventilated preterm lambs would reduce lung injury, and pulmonary and systemic inflammatory responses.

## Materials and methods

### Human amnion epithelial cell isolation and preparation

Human procedures (including the consent process) were approved by the Monash Health Human Research and Ethics Committee (ref #: MUHREC-CF13/2144-2013001109). Placentae were obtained from women with uncomplicated pregnancies who provided written consent before elective caesarean section at term (37–40 weeks). Amnion epithelial cells were isolated as previously described [20]. Cell counts and viability were assessed by trypan blue exclusion prior to and after cryopreservation. For treatment of preterm lambs, hAECs from three donors were thawed and combined, washed, counted and assessed for viability, then resuspended at 30x10^6^ cells/ml in sterile phosphate-buffered saline (PBS) for administration.

### Animal experiments

Animal experimentation was approved by the relevant Monash University Animal Ethics Committee (ref #: MMCA/2012/10).

Experimental animals were obtained from a timed mating program managed by the Monash Animal Research Platform, Monash University. They were transported from an open filed environment to indoor housing (12 h light/dark cycle) in individual pens at least 1 week before any experimental intervention. Ewes remained in constant visual contact of other sheep, had continual access to water and were fed a pelleted diet, supplemented with chaff and/or lucerne hay, twice daily.

For the laparotomy procedure, ewes bearing twins at 126 ± 1 (mean ± SD) days of gestation (term ~147 days) were anaesthetised (2% isoflurane in O2, delivered by positive-pressure ventilation, after induction by IV injection of 20mg/kg sodium thiopentone). A surgical monitor (Surgivet Advisor, Smiths Medical, MA, USA) was used for continual monitoring of ewes’ ECG, heart rate, O_2_ saturation (by pulse oximetry) and end-tidal CO_2_. A protocol was in place for an escalating series of interventions should there be deterioration in any of these physiological indices of wellbeing (culminating in immediate euthanasia for severe respiratory or cardiovascular crisis); however all ewes remained well throughout the procedure.

A laparotomy was performed, the fetus was exposed and the trachea orally intubated (using a cuffed 4.0-mm endotracheal tube), excess lung liquid was drained. A pulse oximeter probe (Masimo, Irvine, CA, USA) was placed on the right forelimb. The fetus then received an intratracheal infusion of 90x10^6^ hAECs (in 3 ml PBS) or vehicle (3ml PBS) and the endotracheal tube was clamped until ventilation onset.

The umbilical cord was clamped and cut. The ewe was immediately killed (by IV injection of 5g pentobarbitone) while anaesthetised. The lamb was weighed, dried and placed in a supine position on an infant warmer (Fisher and Paykel Healthcare, Auckland, New Zealand). Each lamb was connected to a mechanical ventilator (Babylog 8000+ ventilator; Dräger, Lübeck, Germany) for ventilation with warmed (37°C) humidified gas with an initial FiO_2_ of 0.21, a rate of 60 breaths/min and an inspiratory time of 0.3 sec. Peak inflation pressure (PIP) was adjusted to target a tidal volume (V_T_) of 15 ml/kg for 15 min without positive end-expiratory pressure (PEEP), but was limited to 45 cmH_2_O. This ventilation regimen was chosen to induce injury [21], so surfactant was not administered in order not to confound the experimental approach.

The umbilical vein was catheterised for intravenous administration of 90x10^6^ hAECs (in 3 ml PBS) or vehicle (3 ml PBS) within 5 min of delivery, and subsequent infusion of alfaxan (rate of 5–15 mg/kg/h; CenVet, Lynbrook, VIC, Australia) for sedation. We showed previously that combined intratracheal and intravenous administration of 90x10^6^ hAECs (via each route) was most effective for modulation of the fetal pulmonary response to intrauterine inflammation [18]. The umbilical artery was catheterised to continually record arterial pressure and heart rate (DTX Plus Transducer; Becton Dickinson, Singapore: Powerlab; ADInstruments, Castle Hill, NSW, Australia), and for intermittent blood sampling. A pulse oximeter (Masimo, Irvine, CA, USA) was attached to the right forelimb (after removal of hair using small animal clippers) for continual measurement of transcutaneous O_2_ saturation (SpO_2_).

Following the initial 15-min ventilation period lambs were ventilated, targeting a V_T_ of 7 ml/kg, for a further 1 h and 45 min, with 4 cmH_2_O PEEP. Arterial blood gases were measured at 5-min intervals for the first 15 min, followed by 15-min intervals until completion of the experiment at 2 h (ABL30, Radiometer, Copenhagen, Denmark). The inspired O_2_ content was titrated to maintain arterial O_2_ saturation (SaO_2_) between 88–95%. A protocol was in place for immediate euthanasia should lambs’ respiratory or cardiovascular status deteriorate sufficiently but physiological variables for all lambs remained within acceptable ranges: no lamb died or required euthanasia before the end of the 2-h ventilation period.

At the completion of the 2-h ventilation period a final blood sample was then collected for analysis of blood leukocyte phenotype, plasma cytokines, and for T cell proliferation assays. Lambs were killed, with IV pentobarbitone (100 mg/kg; Lethabarb, Virbac Pty Ltd, Australia), for tissue collection. In addition, tissues were collected from unventilated ‘control’ lambs immediately after delivery. These control lambs did not breathe before they were humanely killed as described above.

### Ventilatory and blood pressure analysis

Physiological variables were recorded and analysed using LabChart (ADInstruments). Heart rate (HR), peripheral oxygen saturation (SpO_2_), mean arterial blood pressure (MAP), V_T_ (ml/kg body weight), PIP and PEEP were averaged over 10-s epochs at 5, 10, 15, 30, 45, 60, 75, 105 and 120 min. Lung compliance was calculated: Compliance (ml/cmH_2_O/kg) = (V_T_)/(PIP-PEEP).

### Tissue analysis

Bronchoalveolar lavage (BAL) fluid collected at post mortem [22], was centrifuged to collect cells for phenotypic analysis by flow cytometry (FACS). The right cranial lung lobe was fixed at 20 cmH_2_O with 4% paraformaldehyde (PFA) and processed for light microscopic analyses. Lung tissue was sectioned at 5 μm and stained with haematoxylin and eosin. A total of 15 random high-power fields were scored using an established scale [23] for assessment of airway wall thickness, haemorrhage and epithelial sloughing by an investigator blinded to experimental group. Tissue sections were immunostained to count CD45^+^ cells (MCA2220PE, AbD Serotec, UK), as previously described [24].

Separate lung sections were used to identify cells producing IL-8. These underwent antigen retrieval by heating in 0.01M sodium citrate (pH = 6) in a microwave oven (900W) until simmering, then power output was gradually reduced over 7 min. Sections were cooled, endogenous peroxidase was blocked (0.6% H_2_O_2_ in distilled H_2_O for 20 min) and non-specific binding blocked by incubation in 10% normal goat serum/2% bovine serum albumin (BSA) for 30 min. Slides were incubated with an antibody to ovine IL-8 (mouse anti-ovine IL-8, AbD Serotec MCA1660, clone 8M6, diluted 1:4000 in Dako S0809 antibody diluent) overnight at 4°C, washed (0.1% Tween20 in TBS, 3 x 5-min) and incubated with goat anti-mouse IgG (1:500 in Dako S0809 antibody diluent) for 60 min. Sections were washed before incubation in ABC complex (Vectastain ABC kit; Vector Laboratories) for 45 min. Sections were washed and immunostaining was visualized by incubation with 3,3-diaminobenzidine. Sections were counterstained with hematoxylin. Images from 20 non-overlapping fields (400 x 400 μm) from each section were captured at 20x magnification with Aperio software. Quantification of IL-8 immunohistochemistry was performed using Image-Pro Plus (Media Cybernetics). For each field of view, the number of IL-8^+^ cells was counted, and a mean for each animal was calculated.

The right middle lobe was placed into ice-cold RPMI-10 media (RPMI supplemented with 10% FBS and 1% penicillin/ streptomycin; Life Technologies) for quantification of immune cells by FACS as detailed previously [25]. Samples of spleen and posterior mediastinal lymph node (PMLN) were also collected in RPMI-10 for cell quantification by FACS, and for T cell isolation for proliferation assays. Sections of the right caudal lung lobe were frozen in liquid N_2_ and stored at -80°C until required for quantitative real-time polymerase chain reaction (qRT-PCR) analysis.

### Blood sampling and leukocyte isolation

Blood was collected into heparinised tubes. Plasma and cells were separated by centrifugation. Plasma was frozen at -80°C, and buffy coat cells (leukocytes) collected for T cell proliferation assays [26]. For spleen, PMLN and lung, single-cell leukocyte suspensions were obtained by dissociating whole tissue through a 70-μm cell strainer (BD Biosciences, CA, USA) in complete RPMI-1640; red blood cells were lysed (lysis buffer; 155mM NH_4_Cl, 10mM KHCO_3_, 0.1mM EDTA) and cell number and viability assessed by trypan blue exclusion.

### Proliferation assays

Cells from PMLN, spleen and blood were seeded in triplicate in 96-well plates (2.5x10^5^/well) in complete RPMI-1640 medium alone or in the presence of PMA (Phorbol 12-myristate 13-acetate, 20 pg/ml; Sigma-Aldrich, USA) and ionomycin (I; 800 ng/ml; Sigma-Aldrich). Cells were incubated at 37°C for 96 h, followed by addition of 1 μCi/well of [^3^H]-thymidine (Perkin Elmer, Waltham, MA, USA) for a further 18 h. Cells were harvested onto glass-fibre filter mats (Perkin Elmer) and incorporated radioactive nucleic acids counts performed using a Top Count Harvester (Packard Biosciences, CT, USA), and stimulation index (SI; ^3^[H]-thymidine uptake in stimulated cells relative to media alone) calculated.

### Flow cytometry

BAL cells were stained using single-colour immunofluorescence and analysed on a FACSCanto II flow cytometer (BD Biosciences). Macrophage and lymphocyte populations were gated on forward and side scatter followed by analysis of the fluorescent staining profile [25]. Briefly, cells were incubated with mouse anti-ovine monoclonal antibodies against sheep cell surface molecules CD4, CD8, CD21, CD25, CD44, MHC II and 86D (γδ-T cell), using antibodies detailed elsewhere [25], for 20 min at 4°C and washed with PBS containing 1% BSA, followed by incubation with a fluorescein-conjugated secondary antibody (goat anti-mouse IgG; AlexaFluor 647, Invitrogen) for 20 min at 4°C. Cells were washed again, fixed with 4% PFA prior to acquisition, and analysed using FlowJo software (TreeStar Inc, Ashland, OR, USA).

### Plasma cytokine ELISA

Plasma cytokines were measured by enzyme-linked immunosorbent assay (ELISA), as detailed elsewhere [27, 28]. Briefly, 96-well flat-bottom plates (Nunc) were coated with antibody specific for IL-6 (mouse anti-ovine IL-6; 4B6, Epitope Technologies), IL-10 (mouse anti-bovine IL-10; CC318, Serotec) and TNF (mouse anti-ovine TNF; in-house) and incubated with plasma (diluted 1:1 in PBS/1% BSA/0.05% Tween 20) for 1 h at room temperature. After washing, detecting antibodies for IL-6 (polyclonal rabbit anti-ovine IL-6; in-house), IL-10 (biotinylated mouse anti-bovine IL-10; CC320, Serotec) or TNF (polyclonal rabbit anti-ovine TNF; in-house) were added for 1 h at room temperature prior to incubation with horseradish peroxidase (HRP)-conjugated swine anti-rabbit immunoglobulin (Ig, Dako) for IL-6 and TNF, or streptavidin-HRP conjugated Ig (Dako) for IL-10, for 1 h at room temperature. After further washing, plates were incubated with tetramethylbenzidine substrate solution (TMB, Invitrogen) for 15–20 min in the dark at room temperature. Reactions were stopped with the addition of 2M H_2_SO_4_ and optical density (OD_450_) read (Benchmark Plus^TM^ Microplate reader; BioRad, CA, USA). Recombinant ovine IL-6 (in-house), bovine IL-10 (G. Entrican, Moredun, Scotland) and ovine TNF (in-house) were used for standard curves [27].

### Quantitative real-time PCR (qRT-PCR)

Messenger RNA for ovine IL-1β, IL-6, IL-8, connective tissue growth factor (CTGF), early growth response protein 1 (EGR1) and cysteine-rich angiogenic inducer 61 (CYR61) were measured in lung tissue. Messenger RNA (mRNA) for serum amyloid A-3 (SAA-3), C-reactive protein (CRP) and hepcidin were measured in liver tissue. RNA was isolated using an extraction kit (RNeasy maxi (lung) or midi (liver) kit; Qiagen, VIC, Australia) as per manufacturer’s instructions. Complementary DNA was transcribed using the Superscript III reverse transcription kit (Life Technologies) as per manufacturer’s instructions. Relative gene expression was quantified by qRT-PCR using the housekeeping gene 18S and ΔCt method of analysis. Data are expressed as relative change from the mean level in the unventilated control group.

### Statistical analyses

Data were analysed using one-way ANOVA and Bonferroni *post hoc* analysis, or Kruskal-Wallis ANOVA on ranks and Dunn’s *post* hoc test, to compare unventilated control, and vehicle- or hAEC-treated ventilated groups. When comparing serial data between vehicle- and hAEC-treated groups, a two-way repeated measures ANOVA with Holm-Sidak *post hoc* analysis was used. Where raw data were not normally distributed, they were transformed to achieve normality for analysis. Data were analysed using SPSS v20 statistical analysis software (IBM, Armonk, NY, USA) or GraphPad Prism (GraphPad Software Inc., La Jolla, CA, USA). Data are expressed as mean ± standard error of the mean (SEM).

## Results

### Measurements at birth and responses to ventilation

Numbers of lambs in each group, fetal arterial blood gas status, birth weight and ratio of males to females were similar between groups (Table 1).

**Table 1:** Birth characteristics and arterial blood gas values.

	Control	Vehicle-treated	hAEC-treated
**Male:Female (n)**	4:6	3:7	3:5
**Birth weight (kg)**	3.46 ± 0.39	3.45 ± 0.52	3.33 ± 0.36
**pH_a_**	7.35 ± 0.03	7.34 ± 0.06	7.30 ± 0.06
***P*_aCO2_*(mmHg)*.**	48.0 ± 6.2	46.1 ± 10.4	56.1 ± 7.0
***P*_aO2_*(mmHg)*.**	31.3 ± 13.5	32.5 ± 8.4	31.5 ± 3.0
***S*_aO2_ (%)**	75.6 ± 23.4	82.9 ± 11.4	72.9 ± 6.5

Data are mean ± SEM. pH_a_: arterial pH. P_aCO2_: pressure of arterial carbon dioxide. P_aO2_: pressure of arterial oxygen. S_O2_: arterial oxygen saturation.

**Fig 1:**
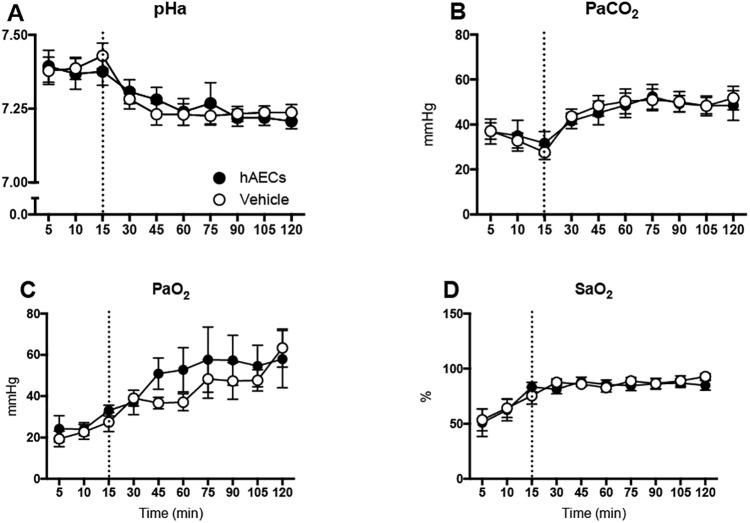
Blood gas measurements. Arterial pH (pHa; A), Partial pressure of carbon dioxide (PaCO_2_; B) and oxygen (PaO_2_; C), and arterial oxygen saturation (SaO_2_; D) of vehicle- (open circles; n = 10) and hAEC-treated (closed circles; n = 7) lambs over 2 h of ventilation. Dotted line represents end of injurious ventilation. Data are mean ± SEM.

**Fig 2:**
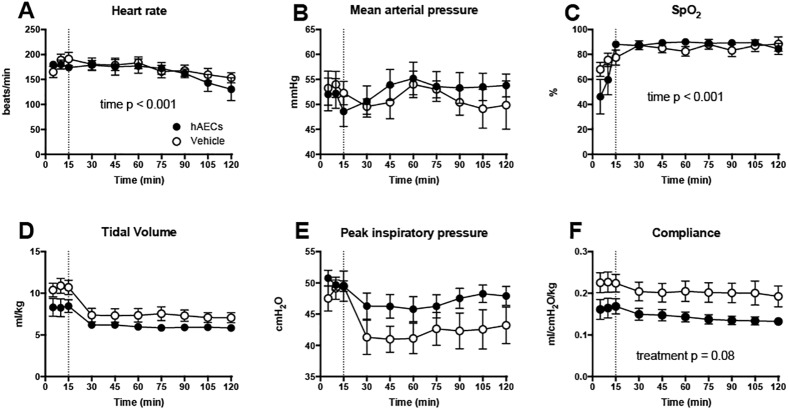
Physiological parameters during ventilation. Heart rate (A), mean arterial pressure (B), peripheral oxygen saturation (C), tidal volume (D), peak inspiratory pressure (PIP; E) and dynamic respiratory system compliance (F) of vehicle- (open circles; n = 10) and hAEC-treated lambs (closed circles; n = 7). Dotted line represents end of injurious ventilation. Data are mean ± SEM.

Over the 2 h ventilation period there were no differences in blood gas parameters (pH, PaCO_2_, PaO_2_ and SaO_2_) between vehicle- and hAEC-treated groups (Fig 1A–1D). Heart rate (Fig 2A) was lower at 120 min than at other times after the onset of ventilation (p < 0.001) in all lambs. SpO_2_, tidal volume, PIP and MAP were not different between vehicle- and hAEC-treated ventilated lambs (Fig 2B–2E). Lung compliance in hAEC-treated lambs tended lower than in vehicle-treated lambs (p = 0.08; Fig 2F).

**Fig 3:**
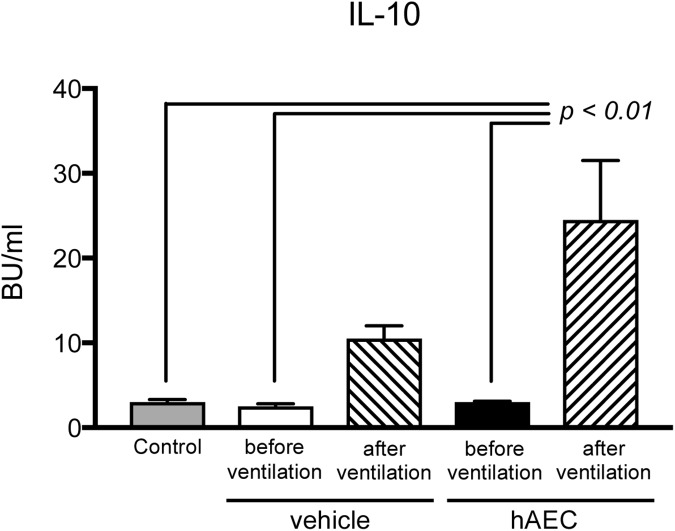
Plasma IL-10 concentrations of unventilated control lambs (grey bars; n = 6), and vehicle- (n = 5) and hAEC-treated (n = 6) ventilated lambs (before and after ventilation). P value refers to comparison with groups indicated by lines. Data are mean ± SEM biological units (BU)/ml (where one BU of ovine IL-10 shows 50% inhibition of IFNγ production in blood cell cultures [29]).

Umbilical arterial plasma concentrations of TNF, IL-6 and IL-10 were comparable between control, vehicle- and hAEC-treated groups before ventilation (TNF and IL-6 data shown in S1 Table). Plasma IL-10 levels at the end of the 2-h ventilation period were higher than before ventilation in the vehicle- and hAEC-treated groups (p<0.001; Fig 3); values tended higher in hAEC-treated lambs than in the vehicle-treated group. Plasma TNF and IL-6 concentrations were not altered by ventilation, and were not different between vehicle- and hAEC-treated groups.

### Characterization of immune cells in BAL

**Fig 4:**
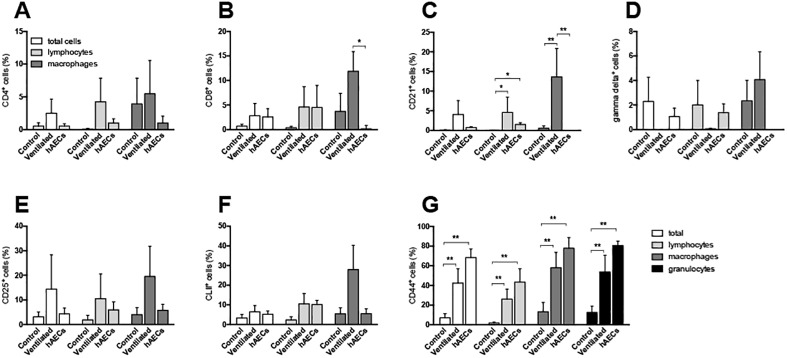
Cell phenotypes in bronchoalveolar lavage fluid samples. CD4^+^ (A), CD8^+^ (B), CD21^+^ (C), γδ^+^ (D), CD25^+^ (E), CLII^+^ (F) and CD44^+^ expression of total BAL cells (white bars), lymphocytes (light grey), macrophages (dark grey) and granulocytes (black bars; panel F only) of control (n = 6), vehicle- (n = 5) and hAEC-treated ventilated lambs (n = 6). ^#^p < 0.05 vs. same cell type in vehicle-treated ventilated group, ^##^p < 0.01 vs. same cell type in vehicle-treated ventilated group, ** p < 0.01 vs. same cell type in control group. Data are mean ± SEM.

Immune cell proportions from BAL samples are shown in Fig 4. The proportion of CD8^+^ macrophages from BAL was higher in vehicle-treated lambs than in hAEC-treated lambs (Fig 4B). Proportions of CD21^+^ lymphocytes and macrophages in BAL were higher in vehicle-treated lambs than in unventilated controls; the proportion of CD21^+^ lymphocytes (B cells), but not macrophages, was higher than control in hAEC-treated lambs (Fig 4C). The percentage of total CD44^+^ cells in BAL, as well as CD44^+^ lymphocytes and macrophages, was greater than control in vehicle- and hAEC-treated lambs, with values tending higher in the hAECs group (Fig 4G). Proportions of CD4^+^ (Fig 4A), gamma delta^+^ (γδ^+^ T cell subpopulation; Fig 4D), CD25^+^ (T cells expressing the IL-2 receptor, a marker of T cell activation; Fig 4E) or MHC II^+^ (Fig 4F) cells in BAL were not different between groups.

### Lung inflammatory gene expression

**Fig 5:**
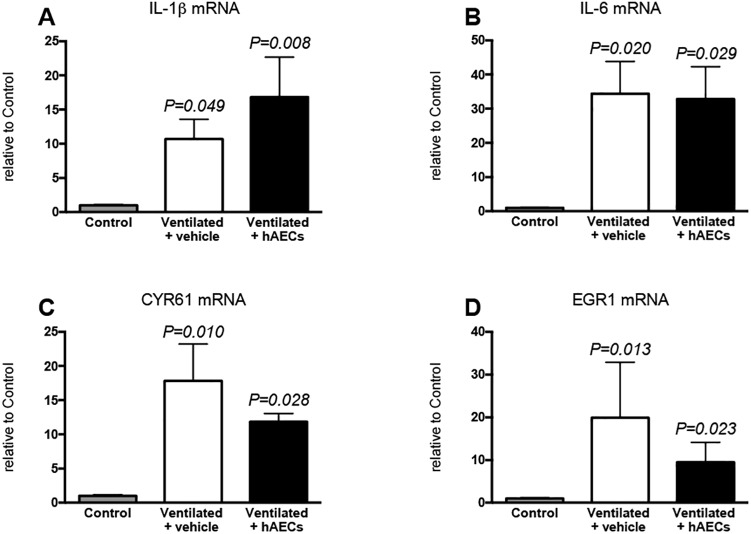
**Lung tissue mRNA levels** of IL-1β (A), IL-6 (B), CYR61 (C) and EGR1 (D) in lung tissue from unventilated control lambs (grey bars; n = 5), and vehicle- (white bars; n = 6) and hAEC-treated (black bars; n = 4) lambs. Data were analysed by one-way ANOVA with bonferroni post hoc analysis: P values relate to comparisons with the control group. Data are mean ± SEM.

**Fig 6:**
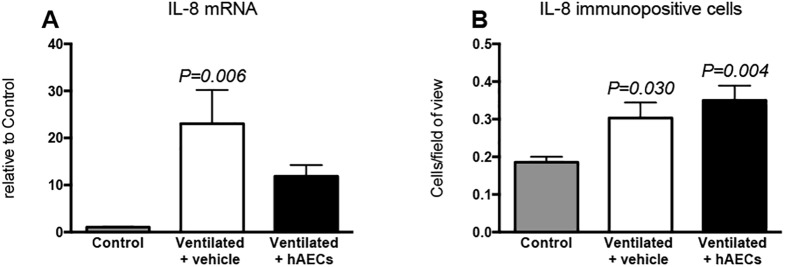
Interleukin (IL)-8 gene and protein expression. Messenger RNA (mRNA) levels (relative to control; A) and numbers of IL-8 immunostained cells in lung tissue from unventilated controls (grey bars; n = 8), and vehicle- (white bars; n = 7) and hAEC-treated (black bars; n = 6) lambs. P values relate to comparisons with the control group. Data are mean ± SEM.

Levels of mRNA for IL-1β (Fig 5A) and IL-6 (Fig 5B) were higher than control in lung tissue from vehicle- (p = 0.049 and p = 0.020 respectively) and from hAEC-treated lambs (p < 0.008 and p = 0.029 respectively), but were not different between these 2 ventilated groups. IL-8 mRNA levels (Fig 6A) were higher than control in vehicle-treated lambs (p = 0.006), but not in hAEC-treated lambs. Levels of mRNA for early lung injury response genes CYR61 (Fig 5C) and EGR1 (Fig 5D) were higher than control in vehicle-treated (p = 0.010 and p = 0.013 respectively) and in hAEC-treated lambs (p = 0.028, and p = 0.023 respectively); there was no difference in these parameters between ventilated groups. Levels of mRNA for CTGF were not significantly different between groups (control, 1.0 ± 0.13; vehicle-treated, 6.7 ± 2.8; hAEC-treated, 8.3 ± 7.9).

### Lung histology and immunohistochemistry

**Fig 7:**
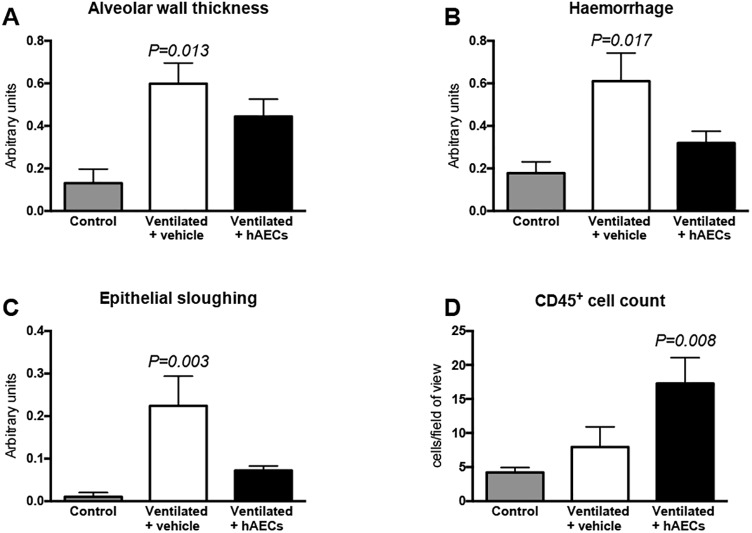
Histological assessments of lung injury. Alveolar wall thickness (A), haemorrhage (B), epithelial sloughing (C), and CD45^+^ cell counts (D) in lung tissue from unventilated controls (grey bars; n = 5), and vehicle- (white bars; n = 8) and hAEC-treated (black bars; n = 6) lambs. P values relate to comparisons with the control group. Data are mean ± SEM.

Alveolar wall thickness (p = 0.013; Fig 7A), haemorrhage (p = 0.017; Fig 7B), and epithelial sloughing (p = 0.003; Fig 7C) scores were higher in vehicle-treated lambs than in unventilated control lambs: scores in hAEC-treated lambs were intermediate between control and vehicle-treated groups.

The numbers of CD45^+^ cells in the parenchyma of the right upper lung lobe examined were higher in hAEC-treated lambs than in vehicle-treated lambs (p = 0.008; Fig 7D). Numbers of CD45^+^ cells in lung tissue from vehicle-treated lambs were intermediate.

Numbers of IL-8^+^ cells were higher in vehicle- and hAEC-treated lambs than in unventilated controls (p = 0.03 and p = 0.004, respectively) but were not different between ventilated groups (Fig 6B).

### Systemic indices of inflammation and immune activation

Hepatic levels of mRNA for SAA-3 were approximately 7-fold higher in vehicle-treated lambs than in unventilated controls and 12-fold higher than in controls in hAEC-treated lambs (p = 0.06 and p = 0.033 respectively; data not shown), but there was no difference between ventilated groups. Hepatic hepcidin and CRP mRNA levels were not different between the 3 groups of lambs (data not shown).

There was no difference in T cell proliferative responses in cells isolated from PMLN, spleen or blood between the 3 groups of lambs (data shown in S2 Table).

## Discussion

Administration of human amnion epithelial cells modulated the acute lung injury and inflammatory response to mechanical ventilation in preterm newborn lambs. This is the first such study in newborn lambs. Our findings extend our previous reports showing that hAECs can moderate *in utero* lung inflammation and tissue remodelling caused by *in utero* ventilation (21) or intra-amniotic LPS injection [18] in fetal sheep, and demonstrate immunomodulation by hAECs newborn preterm lambs *in vivo*.

Although total immune (CD45^+^) cell counts were elevated by hAEC administration, histological indices of lung injury were not different between hAEC-treated lambs and unventilated controls, suggesting that recruited CD45^+^ cells did not cause significant damage to the lungs (at least over the period of our study). The greater infiltration of CD45^+^ cells into the lungs of hAEC-treated lambs is consistent with our observations of the lungs of fetal sheep exposed to LPS *in utero* [18]. These observations suggest that hAECs augment inflammatory cell recruitment in response to lung inflammation and injury. We consider these cells are unlikely to be neutrophils because IL-8 was not elevated, as would be a typical response to neutrophilic infiltration [30, 31]. The reduction in IL-8 mRNA in lung tissue in lambs treated with hAECs likely reflects the increased recruitment of CD45^+^ cells, with the number of IL-8 producing cells remaining constant.

The greater number of CD45^+^ cells in the lungs of hAEC-treated lambs may reflect an increased recruitment of regulatory T cells (T_regs_), consistent with observations in hAEC-treated mice following bleomycin-induced lung injury [32]. Indeed, T_regs_ are critical for hAEC-induced polarisation of macrophages from an M1 to a reparative M2 type phenotype in adult mice with bleomycin-induced lung injury, and a resultant mitigation of lung fibrosis [32]. The anti-inflammatory cytokine IL-10 (we found that endogenous levels tended to be elevated in plasma of hAEC-treated lambs) also plays a role in alternative activation of M2 macrophages [33]. The limited availability of sheep-specific reagents precluded more detailed immune analyses of the neutrophil, M1/M2 macrophage and T_reg_ populations in the lungs of hAEC-treated lambs. Nonetheless, we consider modulation of the local inflammatory response is the most likely mechanism through which hAECs are protective against acute and longer-term lung injury, rather than through reparation dependent on hAEC engraftment, as has been shown in previous studies [17, 18, 34].

We observed hAEC-induced immunomodulation in the lungs of ventilated lambs. CD44 is an adhesion molecule involved in cell-matrix interactions and immune cell trafficking that plays an important role in repair; as demonstrated in a mouse lung inflammation model [35, 36]. In our study, the increase in CD44 expression on BAL cells collected from all ventilated lambs indicates rapid activation of mechanisms for resolution of tissue damage caused during ventilation. Ventilation also resulted in a significant increase in the percentage of CD21^+^ B lymphocytes (CD21 is also known as complement receptor type 2, and is expressed on a subpopulation of B cells in sheep), and both CD8^+^ and CD21^+^ macrophages, which was not observed in BAL collections from the hAEC-treated lambs. CD8 expression by alveolar macrophages [37] and macrophages involved in traumatic tissue responses [38] has been reported previously, and is linked to a pro-inflammatory pathway of macrophage activation involving TNF and IL-1 cytokine secretion [39]. CD21 is commonly expressed by interdigitating dendritic cells within lymph nodes [40], although expression has also been found on airway dendritic cells in sheep [41]. The decrease in BAL cell CD8 and CD21 expression by hAECs demonstrates modulation of airway inflammation in ventilated lambs. Our observations are in agreement with a recent study showing mesenchymal stem cell (MSC)-mediated immune modulation in the airways involving monocytes/macrophages [42].

Administration of hAECs to mice modulates the immune response to decrease long-term fibrosis and BPD-like lung injury [16, 43, 44] and can repair established lung injury [45]. Our study provides evidence for a role of hAECs in reducing lung tissue injury through acute modulation of local inflammatory responses. Additionally, the local pulmonary inflammatory response to mechanical ventilation may induce inflammation and injury in other organs such as the brain [21]. We showed that hAECs modulate ventilation-induced brain inflammation in the lambs from this study [46]. It is possible that the beneficial effects on the brain are due to initial immunomodulation in the lung, which was the primary site of injury in these experiments. Although we observed no sign of systemic immunomodulation within the relatively brief (2 h) period of this experiment, we consider it possible that longer-term immunomodulation may occur as a result of attenuation of inflammation within the lungs.

Our study showed no overt adverse effects of hAEC administration. However, lung compliance tended lower in hAEC-treated lambs than in vehicle-treated lambs. It is possible that tracheal administration of hAECs results in an acute increase in airway resistance, perhaps by clumping of cells in the airways. We recently showed that hAEC function is not impaired *in vitro* by surfactant administration [47]: co-administration might thus provide better dispersal of the cells within the lungs, thus avoiding this effect.

## Conclusions

The amniotic membrane and hAECs are effective and safe for therapeutic use in adults, in whom they have been used mainly for burn treatment and ocular repair for some time [48, 49]. Administration of hAECs, in our current study and in previous experiments undertaken by us, reduces lung injury and fibrotic BPD-like injury through immune modulation. Thus, hAECs may be a viable clinical therapy for neonatal pulmonary injury and prevention of bronchopulmonary dysplasia in preterm human neonates.

## Supporting information

S1 Table Plasma TNF and IL-6 concentrations. (DOCX)

S2 Table Cell proliferation. (DOCX)
