# Supplementary material for: Human amnion epithelial cells modulate the inflammatory response to ventilation in preterm lambs
Source: PLoS One. 2017 Mar 27;12(3):e0173572. doi: 10.1371/journal.pone.0173572 (PMC5367683; doi:10.1371/journal.pone.0173572)
Supplement: S1 Table — (DOCX) [file pone.0173572.s001.docx]

**S1 Table: Plasma TNF and IL-6 concentrations**

|  | Control | Vehicle-treated | hAEC-treated |
| --- | --- | --- | --- |
| TNF before ventilation (ng/ml) | 0.50 ± 0.11 | 0.53 ± 0.22 | 0.45 ± 0.21 |
| TNF after ventilation (ng/ml) |  | 0.62 ± 0.30 | 2.20 ± 1.26 |
| IL-6 before ventilation (ng/ml) | 2.51 ± 0.16 | 2.32 ± 0.15 | 2.38 ± 0.25 |
| IL-6 after ventilation (ng/ml) |  | 2.27 ± 0.12 | 5.33 ± 1.97 |

Data are mean ± SEM. TNF: tumor necrosis factor. IL-6: interleukin-6.
