# Supplementary material for: Human amnion epithelial cells modulate the inflammatory response to ventilation in preterm lambs
Source: PLoS One. 2017 Mar 27;12(3):e0173572. doi: 10.1371/journal.pone.0173572 (PMC5367683; doi:10.1371/journal.pone.0173572)
Supplement: S2 Table — (DOCX) [file pone.0173572.s002.docx]

**S2 Table: Cell proliferation**

| Cell source | Group | | |
| --- | --- | --- | --- |
|  | **Control** | **Vehicle-treated** | **hAEC-treated** |
| PMLN | 3.0 ± 1.1 (3) | 1.2 ± 0.4 (3) | 13.2 ± 5.5 (7) |
| Spleen | 6.7 ± 2.7 (9) | 7.0 ± 2.4 (7) | 4.9 ± 1.3 (8) |
| Blood  Before ventilation |  |  |  |
|  | 33.1 ± 28.6 (7) | 65.5 ± 57.0 (5) | 47.2 ± 21.7 (6) |
| After ventilation | - | 11.8 ± 10.3 (5) | 82.4 ± 36 (6) |

Data are mean ± SEM (n) Stimulation index.
